# Modified external revision-DCR in previous failed endonasal, transcanalicular or external-DCR: technical strategy and teaching Pearls for success

**DOI:** 10.22336/rjo.2023.4

**Published:** 2023

**Authors:** Cem Evereklioglu, Fatih Horozoglu, Osman Ahmet Polat, Hatice Kubra Sonmez, Hidayet Sener, Hatice Arda

**Affiliations:** * Department of Ophthalmology, Division of Oculoplastic, Orbital and Lacrimal Surgery, Erciyes University Medical Faculty, Kayseri, Turkey

**Keywords:** dacryocystorhinostomy, modified, recurrence, revision, technique

## Abstract

**Objective:** To report perioperative findings of patients with multiple failed-dacryocystorhinostomy (DCR) and to determine the success rate of revision external (rEx-DCR) performed by a modified technique.

**Methods:** Thirty-one eyes of 31 patients (19 women, 12 men) with recurrent dacryocystitis or epiphora following at least one previous failed-DCR were assessed regarding the time from initial surgery to recurrence and revision surgery, type of primary surgery (endoscopic, transcanalicular, Ex-DCR), recurrence number, stent usage and the success rate. Relief of epiphora and positive dye test were established as functional and anatomical successes, respectively.

**Results:** The mean age was 43.0 years (8-78), with a mean follow-up period of 21.4 months (6-46). The mean reoperation number was 1.4 (1-5). The mean time from initial surgery to recurrence was 15.2 months (1-55) and to rEx-DCR, 19.8 months (4-65). Untouched medial canthal ligament was observed in 28 (90.3%), improper rhinostomy location in 26 (83.8%), inadequate osteotomy size in 25 (80.6%), single-anterior-flap-only in 5 (16.1%), membranous ostial scar formation in four (12.5%) and no flap in three (9.6%) patients. The success rate was 93.5%, which was lower than our primary modified Ex-DCR (99.1%).

**Conclusions:** The most common reasons for recurrence were small and unsuitable osteotomy locations with intact medial canthal ligaments. “Double-mucosal flap” approach with an anterior sacco-mucosal complex suspension increases the functional success rate, and stent implantation is not obligatory if canalicular problems or small/ atrophic sacs do not exist. The knowledge of technical strategy and teaching pearls improves the success rates of primary and revision surgeries.

**Abbreviations:** DCR = dacryocystorhinostomy, Ex-DCR = external DCR, EE-DCR = endoscopic endonasal DCR, TC-LA-DCR = transcanalicular laser-assisted DCR

## Introduction

Epiphora associated with complete nasolacrimal duct (NLD) obstruction is a common annoying symptom that patients encounter at any age, functionally and socially. Although primary probing may be tried as an initial option for the management of fresh NLD blockages in older children and adults [**1**], the definitive primary treatment of complete obstructions still requires sacco-mucosal anastomosis by conventional dacryocystorhinostomy (DCR) using external (Ex-DCR) [**2**,**3**], endoscopic endonasal (EE-DCR) [**4**,**5**] or transcanalicular laser-assisted (TC-LA-DCR) [**6**] methods with or without silicone stent implantation. 

Among various reasons for surgical failure in traditional Ex-DCR, EE-DCR and TC-LA-DCR, endoscopic evaluations revealed insufficient bone removal, improperly positioned osteotomy site, procedural inability to identify the sac’s localization, adhesions between the anterior and posterior sacco-mucosal flap complexes or the nasal septum, and granulation tissue formation [**2**,**7**].

Although the success rates of surgeries for failed-DCRs are similar between revision (r) rEE-DCR, rTC-LA-DCR and rEx-DCR, numerous published articles have reported endoscopic findings and suggested that revision of failed-DCR cases be performed by rEE-DCR or rTC-LA-DCR surgeries, the results of which have been said to be praised [**8**]. On the other hand, there are only a few papers that report the success rate of rEx-DCR for recurrences. In the present study, perioperative observational findings of rEx-DCR for failed cases were evaluated, a modified suspension technique of the anterior sacco-mucosal flap was used for the first time in revision cases, and the technical strategy with teaching pearls for success is stated.

## Materials and methods

This single surgeon, uncontrolled observational case series was performed in the Department of Ophthalmology. The study followed the principles of the 1964 Declaration of Helsinki. Informed consent was obtained and the research was reviewed and approved by the Ethics Committee of the University (No: 2022/ 78). The surgeon was not responsible for the initial surgeries and all recurrent cases came from other clinics.

From 2002 to 2022, a total of 31 eyes of 31 patients (19 women, 12 men) with unilateral dacryocystitis and epiphora, as a result of recurrent NLD obstruction after primary DCR, were included. An ENT specialist consulted patients to detect possible intranasal pathologies. Exclusion criteria were as follows: patients with endonasal pathologies that affected rhinostomy patency and required surgical intervention, obstructions in the punctum, canaliculus or common canaliculus, and unavailability or lost to follow up postoperatively. Functional and anatomical criteria for success were based on the postoperative resolution of epiphora and patency upon lacrimal syringing. 

The patients with revised Ex-DCR were assessed regarding the time from primary to recurrence and revision surgery, the etiology of primary surgery (EE-DCR, TC-LA-DCR or Ex-DCR), the number of recurrences, silicone stent usage and the success rate of revision surgery at the last visit. Preoperative dacryocystography was not performed in any case and all operations were performed under general anesthesia. 

**Fig. 1:**
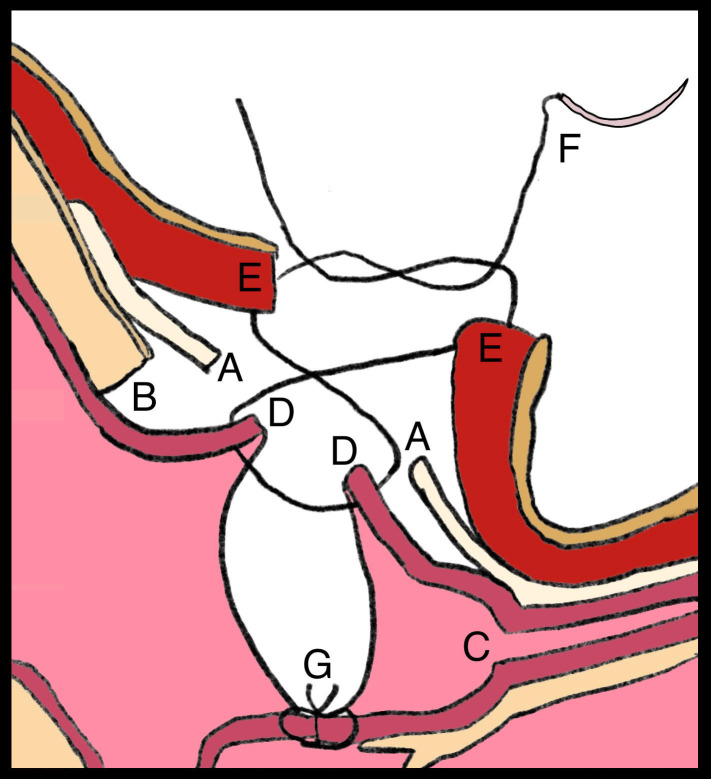
The medial canthal ligament was opened in untouched cases (***A***) to expose the belly of the sac and nearby lacrimal fossa. If the primary osteotomy site was placed improperly, a new bony window (***B***) was created just beneath the medial canthal tendon opposite to the level of the common canaliculus (***C***). New antero-posterior sacco-mucosal flaps were then created (***D***), which were intact in most of the cases. If the osteotomy size was insufficient and smaller than 15 x 15-mm in diameter, it was enlarged in all directions using a Kerrison punch, especially up to the lacrimal fossa superiorly that corresponded again just below the medial canthal ligament (***B***). Anterior sacco-mucosal flaps (***D***) were sutured together up to the orbicularis oculi muscle (***E***) using “figure-of-eight vertical mattress suture technique” (***F***) to prevent collapse back onto the posterior sacco-mucosal anastomosis

All the recurrent cases had a modified surgery by Ex-DCR with “figure-of-eight vertical mattress suture technique” that was described for primary DCR in eyes with complete NLD obstructions [**2**]. Perioperatively, if the medial canthal ligament was found to be untouched, it was opened (**Fig. 1A**) to expose the sac and lacrimal fossa. If the primary osteotomy site was placed improperly, a new bony window was created just beneath the medial canthal tendon (**Fig. 1B**) opposite to the level of the common canaliculus (**Fig. 1C**). Therefore, new anterior and posterior sacco-mucosal flaps were then created in untouched cases (**Fig. 1D**) that corresponded to the belly of the lacrimal sac, which was intact in most of the cases. In cases with untouched medial canthal tendon, it was observed that the osteotomy size was smaller than 10 x 10-mm in all cases. Therefore, if the final osteotomy size was insufficient and smaller than the desired 15 x 15-mm in diameter, it was enlarged in all directions using a Kerrison punch, but especially up to the lacrimal fossa superiorly, that corresponded again just below the medial canthal ligament along with minimal anterior ethmoidectomy. Then, anterior sacco-mucosal flaps (**Fig. 1D**) were sutured together up to the orbicularis oculi muscle (**Fig. 1E**) using the described “figure-of-eight vertical mattress suture technique” (**Fig. 1F**) to prevent collapse back onto the posterior sacco-mucosal anastomosis (**Fig. 1F**). Patients were discharged on the day of surgery with appropriate medication and followed up on the first week and 3-month intervals thereafter.

## Results

The mean age at rEx-DCR was 43.0 (8-78) years and the average time to persistence or recurrence of epiphora following the initial operation was 15.2 months (1-55). The mean period from the first surgery to rEx-DCR was 19.8 months (4-65) and the mean follow-up period was 21.4 months (6-46). Of 31 failed cases, 24 eyes underwent 1 previous surgery, 4 eyes had two, 1 had three, 1 had four and 1 had five (**Table 1**). Therefore, the mean reoperation number was 1.4. Regarding the etiologies of 31 failed-DCRs, four had previous TC-LA-DCR, three had EE-DCR and the remaining 24 had Ex-DCR.

**Table 1:** The main intraoperative findings during revision external dacryocystorhinostomy

	One surgery	Two surgeries	Three, four or five surgeries	Mean
*N* of previous failed surgery	24	4	1 (for each)	1.4
rEx-DCR n= (%)	**Intact MCL** 28 (90.3%)	**Abnormal rhinostomy site** *n*=26 (83.8%) *Inferior* *n*=15 (57.7%) *Anterior* *n*=6 (23.0%) *Infero-anterior* *n*=5 (19.3%)	**Small osteotomy** *n*=25 (80.6%), **Anterior flap-only** *n*=5 (16.1%), **Membranous ostium scar** 4 (12.5%)	**No flap ** 3 (9.6%)
MCL = medial canthal ligament, rEx-DCR = revision external dacryocystorhinostomy				

Overall, the main intraoperative findings during rEx-DCR were found to be an intact-untouched medial canthal ligament in 28 (90.3%) of 31 cases, abnormal rhinostomy location in 26 (83.8%), insufficient osteotomy size in 25 (80.6%), single-anterior flap-only in 5 (16.1%), membranous scarring at the ostium in 4 (12.5%) and no flap in 3 (9.6%) patients. Three or more etiologic factors were determined perioperatively in 20 patients (64.5%) and the narrowest stenotic osteotomy was encountered in TC-LA-DCR. The anterior crus of the intact medial canthal ligament was transected and detached near its periosteal attachment and reflected laterally. The bony wall and the lacrimal sac underneath it were exposed and a new osteotomy was easily performed. Of 26 cases with inappropriate osteotomy location, the ostium was inferior to the lacrimal fossa in 15 eyes (57.7%), more anteriorly in 6 (23.0%) and infero-anteriorly in the remaining 5 (19.3%). Although the anterior part of the maxillary process was removed, the lacrimal bone was found to be intact in these cases that were covering the posterior lacrimal sacs. Therefore, the lacrimal bone and the frontal process of the maxillary bone overlying the lacrimal sac were all removed and minimal anterior ethmoidectomy was performed. In six of the rEx-DCRs procedures, intubation of the silicone stent was performed as a result of atrophic findings in the sac or extreme cicatrization from previous surgeries, which was removed 6 months postoperatively. No cases needed mitomycin-C usage and none of the rhinostomies required a nasal pack at the end of the surgery. Peri- or post-operative complications were not encountered.

Of the re-obstructed 31 failure cases, 29 eyes (93.5%) were successfully treated by our modified Ex-DCR approach and showed resolution of preoperative symptoms and signs with a patent nasolacrimal passage confirmed by syringing at the last examination. The remaining two unsuccessful cases underwent ENT consultation, indicating granuloma and membrane formation at the rhinostomy site. 

## Discussion

Nasolacrimal system blockages distal to the common canaliculus are commonly encountered at the level of mid or lower NLD and reconstructive surgery with DCR in cases of epiphora from their complete obstructions is the basic treatment modality [**1**]. The gold standard in DCR is still an external approach as it has the highest success rate in experienced hands. Indeed, previously published articles of our team, with a total of 504 eyes with epiphora from dacryocystitis, have reported that the success rates after primary surgeries are *(1)* 45.2% for initial probing with or without silicone tube intubation in older children and adults with fresh NLD obstruction [**1**], *(2)* between 87.0% and 88.2% in conventional EE-DCR [**4**,**5**], *(3)* up to 89.8% in traditional Ex-DCR [**3**,**5**], and finally *(4)* 99.1% in modified Ex-DCR [**2**]. However, primary probing with or without silicone tube implantation is not recommended in cases with atrophic sacs, mucocele formation, trauma, acute or long-lasting chronic dacryocystitis, dacryocutaneous fistula and anamnesis of recurrence [**1**].

Endonasal and CT findings revealed that the most common causes for DCR failures are related to smaller intranasal ostium size due to insufficient bone opening during the initial operation along with new bone ingrowth, both of which induce shrinkage of the rhinostomy that reaches 50% of the initial size within the first month following stent removal and up to 35% of the initial size at 1 year postoperatively, resulting in re-obstruction [**9**,**10**]. These patients with previous failed-EE-DCR demonstrated a smaller ostium size when compared with failed-Ex-DCR, all of which were still smaller than the expected 15 x 15-mm diameter. Although the frontal process of the maxillary bone was opened by endoscopic approaches, the anterior ethmoidal bones were not sufficiently removed in most cases (82.6%), which decreased the horizontal dimension of the ostium. In addition, the creation of a posterior flap to cover exposed bones might decrease the incidence of adhesions and bony ingrowth at the newly created NLD ostium following surgery. Indeed, a recent study has reported about a 10-fold recurrence rate in single-flap EE-DCR when compared with the double-flap technique [**11**]. 

In turn, when we externally evaluated the possible etiological factors for recurrent cases, the most prominent perioperative finding was an intact medial canthal ligament with an intact lacrimal bone that covered the supero-posterior aspect of the nasolacrimal sac in 28/ 31 cases, though the frontal process of the maxillary bone was partially removed. Interestingly, both the ligament and the lacrimal bone, just in front of the common canaliculus, were found to be completely untouched even in cases with 3- to 5-time failed-DCR surgery. Surgeons seem to insistently perform the improper technique from the same abnormal lower locations below the medial canthal ligament and, therefore, use the same previously incised sites. However, it is anatomically known that the largest belly of the sac is located just behind the medial canthal ligament and the success rate of Ex-DCR will increase by cutting and detaching the anterior crus of that tendon, which permits sufficient lacrimal bone removal that reaches up to the anterior ethmoidal bones. Because of that reason, it is understandable that the second most common observation in 26/ 31 eyes in the present study was improperly positioned bony ostium that was inferior to the medial canthal tendon and far away from the upper lacrimal fossa but, in turn, close to the NLD entrance. In seven of these cases, the surgical opening was done at a very abnormal position, just anterior to the nasolacrimal sac fossa, near the nasal bone. A true location of the bony ostium, just in front of the common canaliculus and under the medial canthal ligament, is predictive of success. If these rules are met, rEx-DCR will be successful without the need for silicone stent intubation unless there is a (common) canalicular problem or an atrophic sac.

Inadequate lacrimal bone removal was the third most frequent finding in 25/ 31 of failed-DCR patients. It is generally difficult to enlarge the small osteotomy site sufficiently by TC-LA-rDCR or EE-rDCR when compared with rEx-DCR. That is why the success rate of rTC-LA-DCR is known to be lower than that of rEx-DCR. However, in the present article, it was possible to enlarge the inadequately sized and improperly positioned former osteotomy openings by rEx-DCR that was re-positioned and/ or enlarged properly just below the medial canthal ligament near the upper lacrimal sac. Indeed, the size and the proper position of osteotomy are known to be vital factors for surgical success in primary DCR [**12**]. In addition, any granulation tissue was excised easily at the rhinostomy site, if present. 

On the other hand, the importance of flap construction and its number have been stressed in a recent study, which is again vital for preventing recurrence of EE-DCR [**13**]. All our modified revised or primary modified Ex-DCR cases had both anterior and posterior sacco-mucosal flaps that may have contributed to the high success rates. Although some alternative approaches have been suggested for the treatment of failed cases, such as nasolacrimal recanalization (84.4%) [**14**], traditional approaches regarding the success rates of rDCRs revealed that rEE-DCR with silicone tube intubation has been reported to have a success rate of about 82.0% [**15**]. In the present Ex-DCR revision, none of the cases, except for 6 patients with atrophic sacs, required stent implantation, and the success rate was higher with the aid of sufficient bony opening in a proper location and anterior sacco-mucosal suspension. Revision surgeries show 88.2% success in rEE-DCR, 90.5% in TC-LA-rDCR and 93.0% in rEx-DCR [**16**]. It is clear that rEx-DCR still has the highest success rate among the various alternative approaches and our modified rEx-DCR in multiple failed cases is consistent with literature.

In our modified suspension technique for the anterior sacco-mucosal flap complex in primary Ex-DCR [**2**], we recommended that such an approach may be beneficial in cases with small lacrimal sacs or small bony windows and in revision cases, in which induced scarring is a large concern as a result of previous failed surgery. Indeed, we used this technique in all revision cases to keep the anterior nasolacrimal-mucosal flap complex away from the reconstructed posterior flaps that prevent its collapse back onto the posterior anastomosis, which decreases the possibility of mucosal adhesions with nearby tissues and, therefore, fibrosis or granulation tissue formation. For this reason, a larger bony window was first re-created to obtain a sufficient nasal mucosal flap to compensate for the absence of adequate lacrimal sac flap, in which fibrotic reaction or scarring is a large concern, triggered from the previous operation. Therefore, a high success rate of rEx-DCR in the present study, which includes more than 1 revision surgery in some cases, can be attributed to the modified surgical technique that suspends the upper sacco-mucosal complex to the overlying tissues after a properly re-positioned and/ or enlarged bony window was re-created just under the medial canthal ligament. 

The distance between the anterior and posterior sacco-mucosal flaps is another important factor for failures that are especially seen in complicated cases such as small or atrophic sacs, since contraction and scarring of the wound during the healing process aggressively occurs with multiple revision surgeries [**17**]. Therefore, as most proximal nasolacrimal sacs were found to be intact in the present study as a result of the abnormal inferior location of the initial surgical ostium, the use of the “figure-of-eight vertical mattress suture technique” for the suspension of the anterior sacco-mucosal flap may have contributed to the increased success rate of rEx-DCR. Such an anastomosis-suspension technique seems to be impossible to achieve by primary and rEE-DCR or rTC-LA-DCR approaches.

Two articles reported 85.0% success rates for reoperation using rEx-DCR after failed-DCRs. However, the authors used silicone tube intubation with or without mitomycin-C [**8**,**18**]. The success rate of the present paper is higher than both studies. The possible reason is that the small osteotomy was enlarged if they were smaller than 15 x 15-mm, which was 10 x 10-mm in Akçay’s study [**8**]. In addition, the newly created anterior flap-complex was suspended to the overlying tissues to widen the fistulizing pathway. Moreover, silicone tubes were not used in most cases since leaving them may contribute to more granulation tissue formation. Endoscopic or TC-LA-DCR approaches need more surgeon experience with the need of expensive and sophisticated tools, though rEE-DCR has the advantage of less skin scarring along with the chance to treat the nasal anomalies, if present. In turn, revision surgery by external approach needs simple tools and is easy to perform if the critical steps are followed by the surgeon.

## Conclusion

The present article offers detailed external observations of eyes with failed-DCR. Iatrogenic injury from improper technique, especially in an abnormal surgical location, decreases the success rate of this delicate surgery. The results of rEx-DCR are satisfactory if the surgeon knows the physiopathological anatomy of that small area. Therefore, to avoid early or late failure in primary Ex-DCR or to increase the success rate of rEx-DCR, a meticulously designed 20-technical-strategy should be accurately applied for every patient as follows: *1)* the middle turbinate is packed preoperatively with cottonoid strips soaked in vasoconstrictive agents; *2)* a laterally curved skin incision, of at least 1.5-cm-long and 6-8-mm away from the medial canthal angle, is made; *3)* the incision is carried superiorly 4-5-mm above the medial canthal ligament; *4)* the muscle layer is bluntly separated; *5)* the anterior crus of the medial canthal tendon is transected and detached near its periosteal attachment and reflects it laterally so that the largest belly of the lacrimal sac and its nearby fossa can be widely exposed; *6)* the periosteum is vertically (2-mm medial to the anterior lacrimal crest) cut and elevated upward to expose the nasal bone; *7)* the bony window site is selected just below the medial canthal ligament around the lacrimal sac that corresponds to the level of common canaliculus; *8)* the bony window is opened superiorly above the medial canthal ligament and for about 5-6-mm anterior to the anterior lacrimal crest; *9)* the rhinostomy is extended downward to the entrance of the NLD and the frontal process of the maxillary bone, including the whole anterior lacrimal crest, is removed; *10)* the lacrimal bone, including the posterior lacrimal crest, is removed; *11)* minimal anterior ethmoidectomy is performed; *12)* the surgical bony opening is completed for a final diameter of at least a 15 x 15-mm circular window, centering at the lacrimal fossa that stretches out centrifugally; *13)* the lacrimal sac wall is incised vertically from its mid-line, where a lacrimal probe pouches out the medial saccal wall (not anteriorly or posteriorly); *14)* large anterior, but smaller posterior traditional H-flaps of the nasal mucosa are constructed; *15)* both the posterior and the anterior sacco-mucosal flaps are approximated and sutured; *16)* no stent is implanted unless there is a canalicular problem or atrophic sac; *17)* the rhinostomy site should not be packed perioperatively as postoperative bleeding is unusual and collapsing is not expected; *18)* the anterior flap complex is suspended to the overlying orbicularis oculi muscle to keep it from collapsing back into the rhinostomy site and to prevent bone regrowth into the anastomosis site; *19)* the pre-cut medial canthal tendon is repaired with a non-absorbable mattress suture; and finally *20)* the surgery is finished by suturing the skin as usual. 

If applied appropriately, all these aforementioned teaching pearls will improve the surgeon’s learning curve for primary Ex-DCR even in complicated cases with small/ atrophic sacs and revision surgeries. Although the success rates for rDCRs are known to be lower than those found in primary DCRs, modified rEx-DCR may be the gold standard procedure for recurrent cases by the achievement of apposition and suspension of the sacco-mucosal flap. The present article demonstrated that rEx-DCR is a safe and effective method in patients with failed-DCRs and should be considered as a treatment of choice even for eyes with multiple revision surgeries by endonasal or transcanalicular approaches.

**Conflict of Interest statement**

Authors state no conflict of interest.

**Informed Consent and Human and Animal Rights statement**

Informed consent has been obtained from all individuals included in this study.

**Authorization for the use of human subjects**

Ethical approval: The research related to human use complies with all the relevant national regulations, institutional policies, is in accordance with the tenets of the Helsinki Declaration, and has been approved by the Ethics Committee of Erciyes University Medical Faculty, Kayseri, Turkey (No: 2022/ 78).

**Acknowledgements**

None.

**Sources of Funding**

None.

**Disclosures**

None.

## References

[1] Evereklioglu C (2020). Primary probing with and without Monoka silastic stent intubation for epiphora in older children and adults. Curr Eye Res.

[2] Evereklioglu C, Oner A, Somdaş MA, Ketenci I, Dogan H, Mirza E, Ilhan O (2007). Figure-of-eight vertical mattress suture technique for anterior flap suspension to overlying tissues in external dacryocystorhinostomy. Am J Ophthalmol.

[3] Evereklioglu C, Gunduz A, Er H (2000). Comparative results of external dacryocystorhinostomy with or without silicone tube intubation. MN Ophthalmol.

[4] Cokkeser Y, Evereklioglu C, Tercan M, Hepsen IF (2003). Hammer-chiesel technique in endoscopic dacryocystorhinostomy. Ann Otol Rhinol Laryngol.

[5] Cokkeser Y, Evereklioglu C, Er H (2000). Comparative external versus endoscopic dacryocystorhinostomy: results in 115 patients (130 eyes). Otolaryngol Head Neck Surg.

[6] Yoon JM, Nam SW, Woo KI, Kim YD (2018). Transcanalicular diode laser-assisted revision surgery for failed dacryocystorhinostomy with or without distal or common canalicular obstruction. Ophthalmic. Plast Reconstr Surg.

[7] Yu B, Qian Z, Han X, Tu Y, Wu W (2020). Endoscopic endonasal dacryocystorhinostomy with a novel lacrimal ostium stent in chronic dacryocystitis cases with small lacrimal sac. J Craniofac Surg.

[8] Akçay E, Yuksel N, Ozen U (2016). Revision external dacryocystorhinostomy results after a failed dacryocystorhinostomy surgery. Ophthalmol Ther.

[9] Mann BS, Wormald PJ (2006). Endoscopic assessment of the dacryocystorhinostomy ostium after endoscopic surgery. Laryngoscope.

[10] Chan W, Selva D (2013). Ostium shrinkage after endoscopic dacryocystorhinostomy. Ophthalmology.

[11] Bani-Ata M, Aleshawi A, Ahmad M, Saleh O, Ashour R, Khalil H, Alomari S, Alhowary AAA (2020). Endoscopic dacryocystorhinostomy: A comparison of double-flap and single-flap techniques. Ann Med Surg (Lond).

[12] Gokcek A, Argin MA, Altintas AK (2005). Comparison of failed and successful dacryocystorhinostomy by using computed tomographic dacryocystography findings. Eur J Ophthalmol.

[13] Kakizaki H, Kitaguchi Y, Takahashi Y, Mupas-Uy J, Mito H (2016). Prevention of re-obstruction in watery eye treatment: three-flap technique in external dacryocystorhinostomy. Graefes Arch Clin Exp Ophthalmol.

[14] Hong J, Qian T, Wei A, Sun Z, Wu D, Chen Y, Marmalidou A, Lu Y, Sun X, Liu Z, Amparo F, Xu J (2016). Nasolacrimal recanalization as an alternative to external dacryocystorhinostomy for treating failed nasolacrimal duct intubation. Medicine (Baltimore).

[15] Wu S, Xu T, Fan B, Xiao D (2017). Endoscopic dacryocystorhinostomy with an otologic T-type ventilation tube in repeated revision cases. BMC Ophthalmol.

[16] Go Y, Park J, Kim K, Lee S (2015). Comparison of nonlaser endoscopic endonasal revision surgery and diode laser transcanalicular revision surgery for failed dacryocystorhinostomy. J Craniofac Surg.

[17] Çifçi F, Ersanli D, Civelek L, Baloglu H, Karadayi K, Güngör A (2005). Histopathologic changes in the lacrimal sac of dacryocystorhinostomy patients with and without silicone intubation. Ophthal Plast Reconstr Surg.

[18] Ari S, Kürşat Cingü A, Sahin A, Gün R, Kiniş V, Çaça I (2012). Outcomes of revision external dacryocystorhinostomy and nasal intubation by bicanalicular silicone tubing under endonasal endoscopic guidance. Int J Ophthalmol.

